# Emergence of Drug Resistance in Human Immunodeficiency Virus Type 1 Infected Patients from Pune, India, at the End of 12 Months of First Line Antiretroviral Therapy Initiation

**DOI:** 10.1155/2014/674906

**Published:** 2014-04-10

**Authors:** Rajesh T. Patil, Rajiv M. Gupta, Sourav Sen, Srikanth P. Tripathy, Devidas N. Chaturbhuj, Nitin K. Hingankar, Ramesh S. Paranjape

**Affiliations:** ^1^Department of Microbiology, Azeezia Institute of Medical Sciences & Research, Kollam, Kerala, India; ^2^DDGMS (IT) Office of Directorate General of Medical Services (Army) Integrated HQ of MoDAG's Branch, L Block, New Delhi, India; ^3^Department of Microbiology, Army Hospital (Research & Referral), Delhi, India; ^4^National JALMA Institute of Leprosy and other Mycobacterial Diseases (Indian Council of Medical Research), Agra, India; ^5^National AIDS Research Institute (Indian Council of Medical Research), Pune, India

## Abstract

*Introduction*. In India, 4,86,173 HIV infected patients are on first line antiretroviral therapy (ART) as of January 2012. HIV drug resistance (HIVDR) is drug and regimen-specific and should be balanced against the benefits of providing a given ART regimen. *Material & Methods.* The emergence of HIVDR mutations in a cohort of 100 consecutive HIV-1 infected individuals attending ART centre, on first line ART for 12 months, was studied. CD4^+^ T-cell counts and plasma HIV-1 RNA level were determined. * Result.* Out of the 100 HIV-1 infected individuals, 81 showed HIVDR prevention (HIV-1 RNA level < 1000/mL), while the remaining 19 had HIV-1 viral RNA level > 1000/mL. HIVDR genotyping was carried out for individuals with evidence of virologic failure (HIV-1 RNA level > 1000/mL). The most frequent NRTI-associated mutation observed was M184V, while K103N/S was the commonest mutation at NNRTI resistance position. * Conclusion*. Our study has revealed the emergence of HIVDR in HIV-1 infected patients at the end of 12 months of first line ART initiation. For NRTIs, the prevalence of HIVDR mutations was 9% and 10% for NNRTIs. Our findings will contribute information in evidence-based decision making with reference to first and second line ART delivery and prevention of HIVDR emergence.

## 1. Introduction

Estimates place the number of people living with human immunodeficiency virus (HIV) in India in 2009 at 2.39 million with an estimated adult HIV prevalence of 0.31% [1]. Under National AIDS Control Programme (Phase III) of India 300743 patients are on antiretroviral therapy (ART) in 342 ART centers and 650 link ART centers as of January 2012 [1]. ART regimens in resource limited settings are usually selected at the national level following a public health approach. Generally only one first line regimen with alternative regimen(s) incorporating within class drug substitution is available in the public sector [2]. In India, the first line ART consists of zidovudine (AZT)/stavudine (d4T) + lamivudine (3TC) + nevirapine (NVP)/efavirenz (EFZ) [3, 4]. Because of the error-prone nature of HIV replication, its high mutation rate in the presence of drug selective pressure, and because of the need for lifelong treatment, it is anticipated that drug resistant (HIVDR) HIV strains will emerge among persons on treatment even if appropriate ART regimens are provided and optimal adherence to therapy is supported [5]. Emergence of HIVDR is drug and regimen-specific and should be balanced against the benefits of providing a given ART regimen. Studies have shown that the immunological failure can be delayed by 6 months to 2 years from the time of virologic failure [6]. HIV drug resistant mutations in patients on ART can occur before or after virologic failure and can continue to occur and get selected under drug pressure if the failed regimen is not replaced [7]. These drug resistant HIV not only exhibit resistance to several classes of antiretroviral drugs [8] but also cross-resistance within a class of antiretroviral drugs [9]. Due to the high cost of the plasma virus load determination, monitoring of HIV RNA levels in HIV infected patients on first line ART is still not offered as a standard of care test in resource limited settings. Studies have shown that the second line nucleoside reverse transcriptase inhibitor (NRTI) drugs like tenofovir, abacavir, and didanosine and nonnucleoside reverse transcriptase inhibitor (NNRTI) drugs like etravirine will be rendered ineffective if these PLHA harbouring HIVDR mutations are continued on a failing regimen [10]. ART prescription in resource limited settings is population based rather than based on the model of individual patient management utilized in resource-rich nations [2]. In a recent survey on prevalence of transmitted HIVDR among recently infected clients in Mumbai and among newly diagnosed HIV-1 primigravida women in Kakinada, the prevalence of transmitted HIVDR was classified as <5% to all relevant antiretroviral drug classes [11, 12]. This study was carried out to evaluate the emergence of HIVDR in 100 HIV-1 infected individuals at the end of 12 months of initiation of first line ART.

## 2. Material and Methods

### 2.1. Study Participants

This was a cross-sectional study wherein participants consisted of 100 consecutive HIV-1 infected individuals attending the ART centre from 2009 to 2011 who had completed 12-month duration from the time of initiation of first line ART. The first line ART regimen consisted of lamivudine (3TC) + Stavudine (d4T)/Zidovudine (AZT) + Nevirapine (NVP)/Efavirenz (EFZ). After taking written informed consent whole blood was collected in K_3_EDTA. Plasma was separated by centrifugation at 1500 ×g for 10 min at room temperature. The plasma samples were aliquoted and stored in duplicate at −70°C. CD4^+^ T lymphocyte counts were estimated in the whole blood collected in K_3_EDTA by flow cytometry (FACS Count, BD Biosciences Immunocytometry Systems, San Jose, CA). Plasma HIV-1 RNA level was determined by Roche Amp Monitor test, version 1.5 (Roche Diagnostics, Branchburg, NJ).

### 2.2. HIV-1 Drug Resistance Genotyping and Phylogenetic Analysis

**Figure 1:**
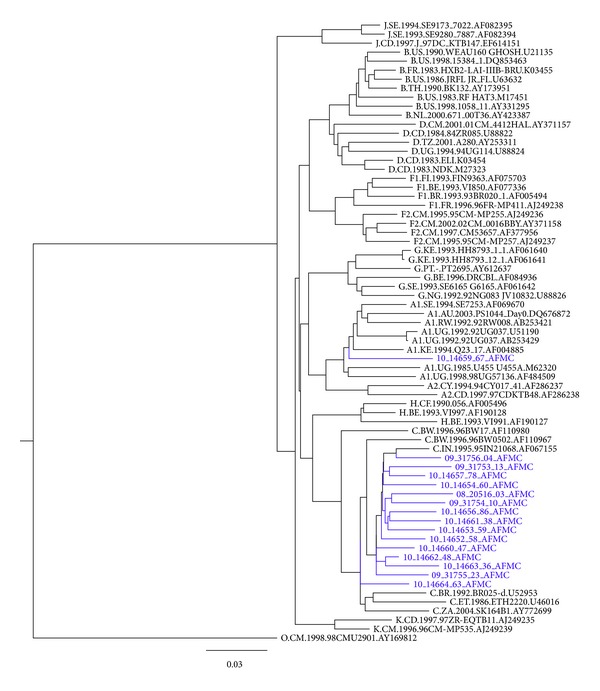
Phylogenetic tree of 16 HIV-1 pol sequences constructed using TreeMaker tool available on Los Alamos HIV sequence database, using general-time-reversible (GTR) distance model.

Plasma samples stored at −70°C were used for detection of HIVDR mutations by in-house assay described earlier after minor modifications [13]. HIV-1 RNA was extracted from plasma using QIAamp viral RNA Mini Kit (Qiagen, Valencia, CA). The HIV-1* pol *gene region encoding protease (PR) and the 5′ end of reverse transcriptase (RT) was amplified by one tube reverse transcription—PCR using the RobusT I RT-PCR kit (Finnzymes Oy, Finland), followed by nested PCR, with outer primers:* Pol 2021F and Pol 4521R. *Two sets of inner primer were used to amplify HIV-1 pol region amplicons of 1–250 base pairs and 1–560 base pairs using* Pol 2135F and Pol 3338R *and* Pol 2135F and Pol 4470R, respectively*. The PCR amplicons were directly sequenced bidirectionally on an ABI PRISM 3100 genetic analyzer system (Applied Biosystems, Foster City, CA). The forward sequencing primers used were* Pol 2041F, Pol 2135F, Pol 2493F, Pol 3012F, Pol 3403F, Pol 3468 *and the reverse sequencing primers were* Pol 2557R, Pol 3117R, Pol 3620R, Pol 3768R, Pol 3999R, Pol 4381R.* The regions sequenced by the in-house assay included PR (1–99) and RT (range: 1–250 to 1–560). The contig sequences were manually edited using SeqScape 2 software (Applied Biosystems) and were submitted to “HIVdb Program: Sequence Analysis” in the Stanford University HIV drug resistance database (http://sierra2.stanford.edu/sierra/servlet/JSierra) for drug resistance interpretation. The phylogenetic tree (Figure 1) was constructed using TreeMaker tool available on Los Alamos HIV sequence database, using general-time-reversible (GTR) distant model (available at http://www.hiv.lanl.gov/) with distance model GTR.

## 3. Results

Among the 100 HIV-1 infected study participants, 74 were males and 26 females. The age range was 21 to 70 years and heterosexual promiscuity was the most common mode of transmission (55%). The various first line ART combinations received by the study participants were (a) 3TC + AZT + NVP (*n* = 53), (b) 3TC + AZT + EFZ (*n* = 14), (c) D4T + 3TC + NVP (*n* = 13), (d) D4T + 3TC + EFZ (*n* = 8), (e) 3TC/D4T + AZT + NVP (*n* = 6), (f) 3TC + AZT + NVP/EFZ (*n* = 4), (g) 3TC/D4T + AZT + NVP/EFZ (*n* = 2).

### 3.1. HIV-1 RNA Levels and CD4^+^ T-Cell Counts

Out of the 100 HIV-1 infected study participants, 81 showed HIVDR prevention (HIV-1 RNA level < 1000/mL), while the remaining 19 had HIV-1 viral RNA level > 1000/mL (range: 1,026–963,000) (median: 7,421). CD4^+^ T-cell count in 10 study participants was >500 cells/µL, 200–499 cells/µL in 64, and 26 had <200 cells/µL. The details of CD4^+^ T-cell counts, HIV-1 RNA level, the first line ART regimen for 19 study participants which had HIV-1 RNA level > 1000/mL are shown in Table 1. Based on the recommendations by WHO to monitor HIVDR prevention and associated factors in sentinel ART sites, individuals showing HIV RNA level (<1000 copies/mL after 12 months of initiation of first line ART) were considered to have “no effective HIVDR mutations” [2, 14] and emergence of HIVDR mutations was suspected in individuals with HIV RNA level ≥ 1000 copies/mL after 12 months of initiation of first line ART.

### 3.2. HIV-1 Drug Resistance Mutations and Subtyping

Of the 19 plasma samples which showed HIV-1 RNA level > 1000/mL, 16 could be amplified and sequenced. Out of these 16 study sequences, 10 displayed one or more HIVDR mutations out of which 9 sequences had NRTI and NNRTI associated resistance mutations and 1 NNRTI associated resistance mutations only (Table 1). No HIVDR mutation was observed in 6 study participants either to NRTI or to NNRTI. The commonest NRTI mutation observed was M184V (8/19) and the most common NNRTI was K103N/S (6/19). None of the study sequences showed any HIVDR mutations in protease region. Out of the 16 sequences 15 belonged to subtype C and 1 to subtype A1.

## 4. Discussion

According to WHO, in HIV-1 infected patients on first line ART for 12 months, the suggested standard to minimize the emergence of HIVDR is ≥70% [2]. In our study the prevention of emergence of HIVDR was 81% while the prevalence of emergence of drug resistance in HIV-1 infected patients at virologic failure on first line ART after 12 months of treatment initiation was 19%. In the previous study, twelve months after ART initiations, 75% and 64.6% achieved viral load suppression of <1000 copies/mL were observed at the Chennai and Mumbai clinics, respectively [15]. Out of the 19 study participants with HIV RNA level > 1000 copies/mL, HIVDR mutations were observed in 10 (52.7%) study participants. No HIVDR mutations were observed in 6 study sequences. Amplification was not successful in 3 plasma samples which had HIV-1 RNA level > 1000 copies/mL. These 9 study participants were classified as having* potential HIVDR* that is specimens with a HIV-1 RNA level > 1000 copies/mL and no evidence of HIVDR on genotype testing [2]. The prevalence of NRTI-associated HIVDR was 9% while it was 10% for NNRTIs. Of these 10 study sequences with HIVDR mutations, 9 exhibited one or more HIVDR mutations for NRTI as well as NNRTI, while the remaining 1 showed NNRTI-associated HIVDR mutation only. Lack of compliance to ART was recorded in 6 study participants. The commonest NRTI mutation observed was M184V (8/19), which was in agreement with previous Indian studies done on ART experienced individuals [13, 15–17]. This M184V HIVDR mutation which is the commonest and earliest HIVDR mutation to occur in lamivudine (3TC) treated individuals causes high-level resistance to lamivudine (3TC) and emtricitabine (FTC) and low-level resistance to didanosine (ddI) and abacavir (ABC) and increased susceptibility to zidovudine (AZT), stavudine (d4T), and tenofovir (TDF) [18]. Thymidine analog mutations (TAMs), selected by the thymidine analogs (AZT & d4T) which decrease susceptibility to these NRTIs, and to a lesser extent to ABC, ddI, and TDF [18], were seen in 4 study sequences. TAMs are common in low-income countries in which fixed-dose combinations containing thymidine analogs are the mainstay of therapy [18]. TAMs accumulate in two distinct but overlapping patterns [18]. The type I pattern includes the mutations M41L, L210W, and T215Y while type II pattern includes D67N, K70R, T215F, and K219Q/E. Type I TAMs were seen in 2 study sequences in our study out of which in 1 study sequence all the three type I TAMs were seen along with D67N. Type II TAMs were seen in 2 study sequences. E44D, an accessory mutation that generally occurs with type I pattern TAM, was seen in 1 study participant. This mutation occurs in about 1% of viruses from untreated patients and in a significantly higher proportion of viruses from patients receiving NRTI [18]. A62V a multinucleoside HIVDR mutation was seen in 1 study sequence. The Q151M complex was not seen in any of the study sequences in our study. N348I a nonpolymorphic HIVDR mutation was seen in 1 study sequence which occurs in about 10% of NRTI-treated patients [18]. N348I causes a twofold reduction in AZT susceptibility when it occurs in combination with multiple TAM [18]. The most common primary NNRTI resistance mutation was K103N/S which was observed in 6 study participants (K103N-5; K103S-1), which is also the most common mutation documented in another Indian study [17], whereas two other Indian studies [13, 16] found Y181C/V & G190A and V106M & Y181C, respectively, as the commonest NNRTI mutations. The other primary NNRTI resistance mutations that were seen in our study were V106M (2/19), Y181C (1/19), Y188L (2/19), and G190A (1/19). All of them cause high-level resistance to nevirapine and variable resistance to efavirenz, ranging from about twofold for V106A and Y181C, sixfold for G190A, 20-fold for K103N, and more than 50-fold for Y188L [18]. L100I, K101P, P225H, F227L, M230L, and K238T are secondary mutations that usually occur in combination with one of the primary NNRTI resistance mutations [18]. Of these secondary mutations P225H (1/19) & K238T/N (2/19) were the secondary NNRTI mutation seen in our study. They usually occur in combination with K103N as observed in our study. These HIVDR mutations synergistically reduce nevirapine and efavirenz susceptibility [18]. M230L, seen in 1 study sequence, may occur alone which decreases the susceptibility of all NNRTI including etravirine by 20-fold or more [18]. V90I was observed in 1 study sequence which is associated with decreased virologic response to etravirine in the DUET clinical trial [18]. This mutation was also documented in earlier Indian studies [13, 19, 20]. Among the study participants with evidence of virologic failure high- level resistance was seen to nevirapine (10/19), lamivudine (8/19), efavirenz (8/19), emtricitabine (8/19), and didanosine (8/19), intermediate resistance to abacavir (4/19), didanosine (4/19), etravirine (3/19), and stavudine (3/19), and low-level resistance to tenofovir (3/19).

In previous HIVDR mutation studies from India on ARV experienced individuals, the median duration of therapy has ranged from 2.15 to 3 years [10, 13, 16, 17, 20]. Almost all ART centers in India monitor HIV infected individuals on first line ART only with CD4^+^ T-cell count which may not give the true picture of response to therapy. This is the first Indian study that has evaluated HIVDR prevention on a population level, 12 months after initiation of first line ART, which can be addressed, if necessary, by changes in ART regimen protocols. Based on the distribution of HIVDR mutations detected in such HIVDR prevention monitoring studies conducted at multiple sites, suitable action can be planned to initiate alternative as well as second line ART protocols. Early recognition of emergence of HIVDR mutations in HIV-1 infected patients on first line ARV for 12 months with HIV-1 RNA level testing followed by HIVDR testing will help in preserving second line drugs and can also delay the need for newer class of antiretroviral drugs which will be costly for resource limited countries like India. In addition, emergence of HIVDR in HIV-1 infected individuals on first line ART can be further reduced if issues like patient related factors and ART site factors are identified and addressed.

In conclusion, to the best of our knowledge, this is the first Indian study carried out to monitor HIVDR mutations in HIV-1 infected individuals after 12 months of first line ART initiation. In India, multicentric monitoring of HIVDR prevention in sentinel ART sites in HIV-1 infected individuals after initiation of first line ART will be an essential component of national ART program, so as to provide necessary data for national working groups in evidence-based decision making with reference to first and second line ART delivery and prevention of HIVDR emergence.

## 5. Sequence Data

GenBank accession numbers for the 16 study sequences reported in our study were from HQ456667 to HQ456682.

## Figures and Tables

**Table 1:** Details of GenBank accession number, first line ART regimen, CD4^+^ T-cell counts, HIV-1 RNA level, and HIVDR mutations of 19 plasma specimens with HIV-1 RNA level > 1000 copies/mL.

Serial number	GenBank accession number	ART Combination	CD4^+^ T-cell counts (cells/*μ*L) after 12 months of ART initiation	HIV-1 RNA level (copies/mL) after 12 months of ART initiation	HIVDR mutations	
					NRTI mutations	NNRTI mutations
1	HQ456679	3TC, AZT, NVP	312	9,63,000	M41L, M184V, T215Y, N348I	Y188L

2	HQ456669	3TC, AZT, NVP, EFZ	184	83,635	D67N, T69ADNT, K70R, M184V, T215F, K219Q	K103N, Y188, M230L

3	HQ456677	3TC, AZT, NVP, EFZ	95	33,668	D67N, K70R, M184V, T215F, K219EKQ	K103N, P225H, K238T

4	HQ456671	3TC, AZT, NVP, D4T	224	22,034	M184MV	K103N

5	HQ456667	3TC, AZT, NVP	<50	13,491	M41L, E44D, D67N, M184V, L210LS, T215Y	A98G, K101Q, Y181C

6	HQ456681	3TC, AZT, NVP, EFZ	240	7,421	M184V	K103N

7	HQ456680	3TC, AZT, EFZ	290	7,310	None	K103N, V106MV

8	HQ456672	D4T, 3TC, NVP	375	2,604	L74LV, M184V	K103S, M23OL, K238N

9	HQ456675	D4T, 3TC, NVP	275	1,397	M184V	G190A

10	HQ456668	3TC, AZT, NVP	368	1,335	M184V	K103N

11	HQ456673	3TC, AZT, NVP	143	4,12,721	None	None

12	HQ456678	3TC, AZT, NVP	280	58,243	None	None

13	HQ456674	3TC, AZT, NVP	264	39,916	None	None

14	HQ456676	3TC, AZT, NVP	278	2,526	None	None

15	HQ456670	D4T, 3TC, NVP	254	2,032	None	None

16	HQ456682	3TC, AZT, NVP	370	1,614	None	None

17	—	3TC, AZT, NVP	113	3,69,247	No amplification	

18	—	3TC, AZT, NVP, EFZ, D4T	390	2,030	No amplification	

19	—	3TC, AZT, NVP	240	1,026	No amplification	
